# Blood pressure variability estimated by ARV is a predictor of poor short-term outcomes in a prospective cohort of minor ischemic stroke

**DOI:** 10.1371/journal.pone.0202317

**Published:** 2018-08-24

**Authors:** Zefeng Tan, Heng Meng, Dawei Dong, Ying Zhao, Anding Xu

**Affiliations:** Department of Neurology, the First Affiliated Hospital, Jinan University, Guangzhou, Guangdong, China; National Yang-Ming University, TAIWAN

## Abstract

Prior studies have shown that patients with minor ischemic stroke have substantial disability rates at hospital discharge. We sought to determine whether blood pressure variability (BPV) estimated by average real variability (ARV) is one of the predictors of poor outcome at 90 days. Four hundred fifty-one consecutive patients with ischemic stroke treated within 7 days after onset were enrolled prospectively. Baseline magnetic resonance imaging (MRI) was performed on all subjects. Blood pressure was measured for all recruited patients every 2 hours in the first 24 hours after admission, followed by measurements collected every 4 hours from day 2 to day 7 after admission. ARV was used to estimate BPV. A total of 192 patients with minor ischemic stroke were enrolled, and 11 of them (5.7%) had poor outcomes. Univariate regression analysis showed that early neurological deterioration (X^2^ = 21.44, P = 0.000), severe symptomatic large artery stenosis or occlusion (X^2^ = 9.260, P = 0.000), large artery atherosclerotic stroke (X^2^ = 7.14, P = 0.002), total cholesterol (TC), and D2-7 SBP-ARV (t = 5.449, P = 0.001) of the poor outcome group were significantly higher than those of the good outcome group. Multivariate logistic regression analysis showed that early neurological deterioration (OR 4.369, 95% CI 3.54, 15.65; P = 0.001), severe symptomatic large artery stenosis or occlusion (OR 5.56, 95% CI 3.56, 13.65; P = 0.000), large artery atherosclerotic stroke (OR 3.56, 95% CI 1.45, 7.48; P = 0.004), and D2-7 SBP-ARV (OR 3.96, 95% CI 1.90, 20.18, P = 0.008) were significantly related to poor outcomes. In conclusion, approximately 5.7% of minor ischemic stroke patients had poor outcomes. D2-7 SBP-ARV, early neurologic deterioration, severe symptomatic artery stenosis or occlusion, and large atherosclerotic stroke were the independent risk factors of poor short-term outcomes.

## Introduction

Minor ischemic stroke by definition represents the strokes that only cause minor symptoms or only result in minor neurologic defects, although the definition varies [1]. In real clinical practice, we have found that so-called minor ischemic strokes may be misidentified as such due to minor symptoms. Some clinical studies have recognized that ischemic stroke patients with fast recovery or minor symptoms eventually developed morbidity and mortality [2–4]. Most recent studies of minor stroke have shown that 15% of minor stroke patients (National Institutes of Health Stroke Scale (NIHSS) of 0–3 within 72 hours) had a poor clinical prognosis [5].

Recent studies [6–9] have shown that BPV is one of the main predictors of any prognosis. However, the predictive value of BPV during the acute phase of minor ischemic stroke is uncertain. A recent systematic review of seven studies on the effect of BPV on functional outcome (death or disability) suggested that systolic BPV was significantly associated with a poor functional outcome (pooled odds ratio per 10-mm Hg increment, 1.2; 95% CI (1.1–1.3)) [10]. However, the review recommended future prospective studies to investigate best practices to measure and define BPV in acute stroke as well as to determine its prognostic significance [10]. Various indexes, such as standard deviation (SD), coefficient of variation (CV), the SD over 24 hours weighted for the time interval between consecutive readings (SD24), the average of the daytime and nighttime SDs weighted for the duration of the daytime and nighttime intervals (SDdn), variation independent of the mean (VIM), weighted standard deviation (wSD), and ARV, have been used to evaluate BPV by different researchers, but uncertainty remains about which index should be used to gauge the value of BPV as a risk factor.

The aim of this prospective study was to assess the predictive value of BPV estimated by ARV and the short-term functional outcome in acute minor ischemic stroke patients.

## Materials and methods

We prospectively evaluated consecutive patients with acute ischemic stroke. The study was approved by the ethics committee of the Jinan University. All participants and/or caregivers gave their informed written consent according to the Declaration of Helsinki. All the patients were admitted to the neurological intensive care unit (NICU) in the first 24 hours after admission. After 24 hours observation in the NICU, patients were transferred out of the NICU according to the decisions of clinical doctors. Cerebral computerized tomography (CT) or MRI and magnetic resonance angiography (MRA) were performed and described routinely within 24 hours after admission. Minor ischemic stroke was defined as an NIHSS score < 4.

**Inclusion criteria**: Patients were chosen if (1) their cases conformed to the diagnostic criteria of the 2005 China cerebrovascular disease prevention and treatment guidelines for ischemic stroke; (2) the time of stroke onset was not more than 7 days prior to inclusion; and (3) their NIHSS score at admission was less than 4.

**Exclusion criteria**: Patients were excluded if they (1) were below 18 years of age; (2) had inadequate BP measurements; (3) had other serious or life-threatening diseases before stroke onset; (3) received thrombolytic therapy; or (4) had a previous stroke (modified Rankin Scale score, MRS >2). The research protocol was approved by the medical ethical committee of the First Affiliated Hospital, Jinan University for clinical research, and all the data were analyzed anonymously.

### Measurement of blood pressure

The casual supine blood pressure (BP) was measured in the non-paralyzed arm using a standard electronic sphygmomanometer (OMROM-HEM-7200) upon admission. In the neurological intensive care unit (NICU), BP was measured at 2-hour intervals during the first 24 hours by using a noninvasive BP monitoring device (BeneView T5) and then every 4 hours, up until 7 days after admission, by using a standard electronic sphygmomanometer (OMROM-HEM-7200) by a trained nurse, with 1-hour error in the daytime and 2-hour error in the nighttime. All BP records were entered manually into the electronic medical record (EMR) system. The BP profiles during the first 24 hours and days 2–7 (D2-7) were described using the following parameters for both systolic blood pressure (SBP) and diastolic blood pressure (DBP): standard deviation (SD) and coefficient of variation (CV). In addition, we used average real variability (ARV) [11] to represent BPV. The ARV averages the absolute differences of consecutive measurements and accounts for the order in which the blood pressure measurements are obtained. ARV was calculated by the following formula:

$$\text{ARV}=\frac{1}{\text{N}-1}\sum_{\text{k}=1}^{\text{n}-1}\times \left|{\text{BP}}_{\text{K}+1}-{\text{BP}}_{\text{K}}\right|,$$

where k ranges from 1 to n-1, and n is the number of blood pressure readings.

### Stroke risk factors

Patients with a clinical diagnosis of acute stroke were recruited from admissions to the First Affiliated Hospital of Jinan University. Each subject was assessed and examined according to the Oxfordshire Community Stroke Project (OCSP) classification [12]. Data of functional outcomes at 90 days and initial NIHSS scores were collected along with other relative stroke risk factors, including age, gender, history of hypertension (BP ≥ 140/90 mm Hg according to the World Health Organization-International Society of Hypertension guidelines[13] or presently under treatment with antihypertensive drugs) history of diabetes mellitus (fasting plasma glucose level ≥ 7.0 mmol/l, plasma glucose ≥ 11.1 mmol/l two hours after a 75 g oral glucose load as in a glucose tolerance test, symptoms of hyperglycemia and casual plasma glucose ≥ 11.1 mmol/l, glycated hemoglobin (Hb A1C) ≥ 6.5%[14,15], or under hypoglycemic treatment), history of hyperlipidemia (total cholesterol concentration > 6.22 mmol/L (240 mg/dL), triglycerides>2.26 mmol/L, HDL-cholesterol< 1.14 mmol/ the day after admission or already under lipid lowering therapy for hyperlipidemia), history of symptomatic ischemic heart disease (proven myocardial infarction, history of angina, existence of multiple lesions on thallium heart isotope screen or evidence of coronary disease on coronary angiography), atrial fibrillation (documented during hospitalization or history of atrial fibrillation), current cigarette smoking, alcohol abuse (>50 g per week) and previous stroke. In all subjects, the diagnosis of severe symptomatic intracranial artery stenosis or occlusion was confirmed by MRI angiography (MRA). MRA was performed using a standard protocol of three-dimensional time-of-flight sequences. Symptomatic large artery stenosis was defined as a reduction in luminal diameter of at least 50% in the intracranial arteries and the extracranial carotid artery using the methods described by the Warfarin-Aspirin for Symptomatic Intracranial Disease Study [16] and the North American Symptomatic Carotid Endarterectomy Trial method [17], respectively. The extracranial vertebral artery was not included in the data analysis since MRA cannot be used to clearly differentiate a normal variant of congenital hypoplasia from an acquired stenosis.

### Assessment of outcome

The neurological severity on admission and at 7 days (or at discharge if earlier) was assessed by the NIHSS score by trained stroke neurologists. Early neurological deterioration was defined as an increase of ≥ 3 scores in the NIHSS Scale during the first 7 days after admission. Functional outcome was assessed by the mRS score at 90±7 days after the treatment and was dichotomized into good outcome (mRS 0 to 1) or poor outcome (mRS 2 to 6, death was graded 6). On admission (1^st^ day) and on the 90^th^ day, modified ranking scale was evaluated.

### Statistical analysis

All analyses were performed using SPSS for Windows, release 19.0 (SPSS Inc., Chicago, IL, USA), and a P value of <0.05 was considered as statistically significant. Quantitative variables were summarized as the mean±SD, categorical variables as frequency distribution. The values are presented as the mean±SD, median (interquartile range [IQR] for continuous variable, or as the number (%) of subjects for categorical variables. Comparisons of baseline characteristics and BPV parameters between patients of good outcome and poor outcome were conducted with Pearson *χ*^2^ test, Mann–Whitney U test, or Student *t* test as described. We analyzed the association between the clinical outcomes and relevant covariates with logistic regression analysis adjusting age, gender, hypertension, diabetes mellitus, dyslipidemia, atrial fibrillation, coronary heart disease, previous stroke, current or previous smoking, moderate or heavy alcohol, dual antiplatelet and statin therapy after admission, severe symptomatic artery stenosis, NIHSS at admission and relative blood pressure variables include SD, CV, and ARV of first 24 hours and D2-7. For ARV analysis, patients were divided into low (≤ median) and high (>median) groups using the median value of systolic and diastolic ARV during the first 24 hours and over the subsequent 2–7 days. The patient proportion with the poor outcome was calculated, and its correlation with ARV was assessed using a Pearson *χ*^2^ test. All parameters with statistical significance (p<0.05) in the univariate analysis were introduced into a multivariate logistic regression model to explore predictors for poor outcome. Due to the possible collinearity of tested variables, a stepwise logistic regression model was used.

## Results

**Fig 1:**
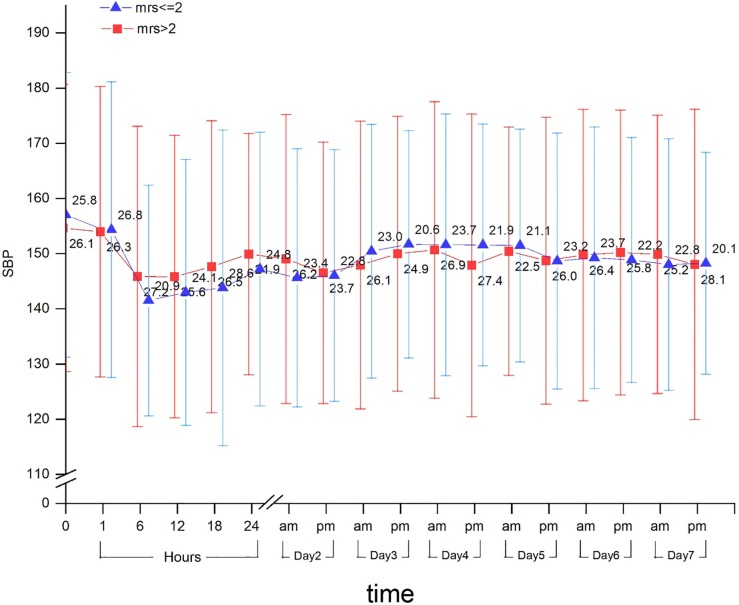
Within-group mean systolic blood pressure and standard deviation of systolic blood pressure over time. Note, the axes are broken.

Of 451 patients (seen in Tables A-F in S1 File), 208 presented with minor strokes. The 90-day outcome analysis was limited to 192 patients after excluding those with missing blood pressure data (n = 13) or recombinant tissue-type plasminogen activator treatment (n = 3). The final analysis included 192 patients. Their age ranged from 33–93, and 24.2% of them were female. The median score of baseline NIHSS was 1 (1–3). At 90 days, 181 patients (94.3%) had good outcomes, and 11 patients (5.7%) had poor outcomes. In total, 18 cases of early neurological deterioration were found (12 and 6 patients in the good outcome and poor outcome group, respectively). Fifty-four cases (28%) were found to have severe symptomatic artery stenosis. Each enrolled patient was monitored for at least 46 time points (10 times in the first 24 hours and 36 times in the next 7 days). The systolic blood pressure in participants in both the good outcome and poor outcome groups fell from days 1 to 7, with the steepest decrease in the first 1 to 6 hours after enrollment (Fig 1). However, we detected no significant differences between the systolic blood pressure and the clinical outcome in either group. Comparisons of baseline characteristics between the two groups are reported in Table 1. Poor outcome results were significantly associated with higher D2-7 SBP-ARV (p = 0.001), early neurological deterioration (6.6% vs 54.5%; p = 0.00), higher TC (3.76±1.45 vs 5.04±1.36; p = 0.004), large artery atherosclerotic stroke (29.2% vs 72.7%; p = 0.002), and severe symptomatic artery stenosis or occlusion (25.4% vs 72.7%; p = 0.000). For the univariate analysis of systolic or diastolic ARV, patients were divided into groups with low and high ARV. A significant association was demonstrated between high D2-7 SBP-ARV and poor outcome (P = 0.005) (Table 2). After adjusting for the known baseline predictors, D2-7 SBP-ARV, symptomatic artery stenosis, major atherosclerosis, and early neurology deterioration were associated with good outcomes at day 90 (Table 3).

**Table 1:** Comparisons of baseline characteristics according to a 3-month functional outcome based on mRS score.

Characteristics	Good outcome	Poor outcome	P
Age, year ± SD	67.3± 9.8	69.9± 8.2	0.205
Female, n (%)	47 (25.9)	4 (36.4)	0.448
Baseline pressure (mmHg, $\bar{x}\pm s$)			
SBP(mmHg)	156.2±25.8	155.42±27.51	0.873
DBP(mmHg)	88.24±15.74	88.52±24.26	0.931
Entry NIHSS (median)	2	2	0.222
Early neurological deterioration, n (%)	12 (6.6)	6 (54.5)	0.00
Smoking, n (%)	85 (47.0)	6 (54.5)	0.624
Alcohol, n (%)	11 (6.0)	1 (9.1)	0.688
History of stroke, n (%)	10 (5.5)	2 (18.1)	0.092
Coronary heart disease, n (%)	22 (12.1)	3 (27.2)	0.148
TC	3.76±1.45	5.04±1.36	0.004
Atrial fibrillation, n (%)	27 (14.9)	2 (18.1)	0.769
Diabetic mellitus, n (%)	48 (26.5)	3 (27.3)	0.956
Hypertension, n (%)	105 (58.0)	8 (72.7)	0.336
Large artery atherosclerotic stroke, n (%)	53 (29.2)	8 (72.7)	0.002
Small vessel disease, n (%)	89 (49.1)	3 (27.2)	0.158
Embolism, n (%)	10 (5.5)	1 (9.1)	0.360
Unclassified, n (%)	20 (11)	1 (9.1)	0.839
Dual antiplatelet, n (%)	126 (69.6)	10 (90.9)	0.131
Statin therapy, n (%)	180 (99.4)	8 (72.7)	1.697
Severe symptomatic artery stenosis or occlusion, n (%)	46 (25.4)	8 (72.7)	0.000
24-h SBP-SD (mmHg, $\bar{x}\pm s$)	13.27±5.27	12.93±5.49	0.669
24-h SBP-CV	0.09±0.04	0.09±0.04	0.772
24-h SBP-ARV (mmHg, $\bar{x}\pm s$)	9.53±2.13	11.02±5.73	0.051
24-h DBP-SD (mmHg, $\bar{x}\pm s$)	10.88±5.14	11.69±5.49	0.3
24-h DBP-CV	0.17±0.07	0.2±0.09	0.14
24-h DBP-ARV (mmHg, $\bar{x}\pm s$)	7.43±3.44	9.85±3.78	0.454
D_2-7_ SBP-SD (mmHg, $\bar{x}\pm s$)	14.22±4.28	14.62±3.93	0.535
D_2-7_ SBP-CV	0.1±0.03	0.1±0.02	0.457
D_2-7_ SBP-ARV (mmHg, $\bar{x}\pm s$)	11.98±3.73	16.21±3.67	0.001
D_2-7_ DBP-SD (mmHg, $\bar{x}\pm s$)	9.17±2.86	9.46±2.71	0.497
D_2-7_ DBP-CV	0.11±0.03	0.12±0.03	0.598
D_2-7_DBP-ARV (mmHg, $\bar{x}\pm s$)	6.56±3.14	8.34±5.60	0.256

**Table 2:** Univariate analysis of patients with high and low systolic and diastolic ARV between good and poor outcomes groups.

	Good outcomes (n = 181)	Poor outcomes (n = 11)	*P*
24-h SBP-ARV			0.756
Low (≤11.0)	90 (49.7)	6 (54.3)	
High (>11.0)	91 (50.3)	5 (45.7)	
24-h DBP-ARV			0.097
Low (≤7.8)	96 (52.8)	3 (27.2)	
High (>7.8)	85 (47.2)	8 (72.8)	
D_2-7_ SBP-ARV			0.046
Low (≤11.8)	105 (58.0)	3 (27.2)	
High (>11.8)	76 (42.0)	8 (72.8)	
D_2-7_ DBP-ARV			0.222
Low (≥7.8)	100 (50.8)	4 (36.4)	
High (>7.8)	81 (49.2)	7 (63.6)	

**Table 3:** Final stepwise logistic regression model to predict poor outcomes.

Variable	*P*	Adjusted OR (95% CI)
D_2-7_ SBP-ARV	0.008	3.96 (1.90, 20.18)
Symptomatic artery stenosis	0.000	5.56 (3.56,13.65)
Major atherosclerosis	0.004	3.56 (1.45,7.48)
Early neurology deterioration	0.001	4.369 (3.54,15.65)

SBP, systolic blood pressure; ARV, average real variability (Final stepwise logistic regression model to predict poor outcome at 90 days, included variables: D2-7 SBP-ARV, Symptomatic artery stenosis, Major atherosclerosis, Early neurology deterioration and TC).

## Discussion

One of the advantages of this study is that it focuses on the influence of read-to-read BPV rather than visit-to-visit BPV. Previous studies have already reported that the assessment of visit-to-visit BPV is a reliable predictor of stroke recurrence [18,19] and of the extent of target organ damage [11,20–23]. Meanwhile, the effect of read-to-read BP variability during the acute phase of stroke has been investigated in a limited number of studies [24–26]. This study is one of the few studies that shows an association between short-term SBP-ARV and clinical functional outcomes. In our study, D2-7 SBP-ARV (OR 3.96, 95% CI 1.90–20.18, P = 0.008) significantly associated with the 3-month functional outcome even when analyzed independently from DBP ARV. While a direct relationship between sub-acute phase BPV and outcome in a large population of stroke patients has been described [6], we still lack evidence from large scale prospective studies.

Second, the relationship between short-term BPV and acute minor ischemic stroke is unclear. Previous large scale retrospective studies mainly focused on cohorts of hypertension or secondary stroke prevention, not minor ischemic stroke patients, and BPV was determined to be a strong predictor of future stroke events [6,7,11,19,27–30]. Other research has also shown that acute phase BPV after stroke was significantly related to the stroke in progression [31–34]. The progression of stroke directly caused poor short-term outcomes. A registry study [34] found that higher post-stroke BP levels (the average values during the 48 hours after onset) were significantly associated with a lower probability of good neurological recovery, elevated risk of neurological deterioration and poor functional outcome. The Glycine Antagonist in Neuroprotection International Trial (GAIN) [32] retrospectively analyzed data in 1445 cases of ischemic stroke patients and showed that variables describing the course of BP over the first 2.5 days have a marked and independent relationship with 1- and 3-month outcomes.

There can only be speculation about the mechanisms underlying this finding. The high BPV may be associated with large vessel atherosclerosis or decreased baroreflex sensitivity in carotid bifurcation [35]. In normal conditions, systolic and diastolic blood pressures change in parallel while responding to physiological stimuli, such as exercise or arousal. In subjects with stiff arteries, when systolic blood pressure increases, often diastolic blood pressure increases less or even falls [36,37], giving rise to larger variability. On the other hand, during the acute phase of stroke, cerebral auto-regulation is impaired and blood flow becomes completely dependent on systemic BP. For this reason, fluctuations in BP may be detrimental for ischemic territories, particularly in the presence of an anatomic or functional compromise of small cerebral vessels [24,38].

As measures of short-term reading-to-reading blood pressure variability, we used the SD, CV and ARV. Various parameters can capture short-term blood pressure variability over 24 hours, but most studies only considered the SD of systolic, diastolic, or both blood pressures.[39–41] Currently, no specific parameter has been selected as the optimum choice to estimate short term BPV in acute stroke. The variability is commonly quantified as SD. This variability index has a notorious shortcoming: it only reflects the dispersion of BP measurements around a single value (the mean) not accounting for the order in which the BP measurements were obtained. SD and CV ignore the order of blood pressure data; as a result, two subjects with different BP measurement sets may have the same SD or CV. An explanation for apparently contradictory results in previous studies may be the selection of the index [standard deviation (SD)] used for quantifying variability[42–43]. ARV was a better predictor than SD24 and SDdn, probably because subjects with different blood pressure profiles might have similar SDs but a different ARV. Thus, ARV might be a more specific measure of blood pressure variability than SD[44]. A proper selection of the variability index is critical to assessing the value of BPV as a risk factor and could explain apparently contradictory results previously reported; a time series variability index such as ARV should be used.[45] ARV, which essentially averages the absolute differences of consecutive measurements, is sensitive to the individual BP measurement order, contrary to the SD. ARV index is a more reliable representation of time series variability than SD and is even more sensitive to the relative high sampling frequency of blood pressure monitoring as in this study[45].

In addition, 208 of 451 patients (46.1%) presented with minor ischemic stroke (NIHSS<4) in this cohort of ischemic stroke. This result is similar to the rate in the Chinese national stroke registry research (30%) [46]. At day 90, 5.7% of them had a poor outcome, which is significantly lower than reported data in China [47]. The possible explanation is that in clinical practice of our center, up to 70% of minor ischemic stroke patients receive dual antiplatelet therapy. The CHANCE (Clopidogrel and Aspirin versus Aspirin Alone for the Treatment of High-risk Patients with Acute Non-disabling Cerebrovascular Event) [48]study has shown that the addition of clopidogrel to aspirin reduced the relative risk of recurrent stroke at 90 days by 32% in Chinese patients with acute minor ischemic stroke or TIA. Furthermore, almost 99% of patients in this study had been prescribed statin therapy. Additionally, compared with data from other stroke registries, we found a trend of a gradual decrease of poor outcomes over time (The Harvard Cooperative Stroke Registry (1978) 20%[49], Lausanne Stroke Registry (1988) 34%[50], the China National Stroke Registry (2008) 14.5%[47], and the Austrian Stroke Unit Registry (2010) 4.5%[51]). The recognition and application of recent guidelines have remarkably improved the outcomes of patients.

There are also some limitations in this study. First, the baseline NIHSS scores were acquired within 7 days of stroke, which is longer than in other studies. For this reason, our cohort may represent a more stable minor ischemic stroke group. Moreover, there was no specific analysis of the different items of the NIHSS score. Furthermore, this is a small sample study conducted in a single-center, and our findings carry a risk of causality error.

## Conclusion

This study is one of the few prospective studies that uses ARV as a predictor to determine functional outcomes. Future large scale prospective studies on the relationship between ARV and minor ischemic strokes are warranted.

## Supporting information

S1 File Supporting tables. Table A. Frequency data of 474 cases of ischemic stroke patients Table B. Data of age, NIHSS (24hours after admission) and biomarkers. Table C. Data of time from onset to admission. Table D. Data of first Blood pressure after admission. Table E. Data of Blood pressure variability (24hours after admission). Table F. Data of Blood pressure variability (D2 to D7 after admission).
(DOCX)
