# Supplementary material for: Blood pressure variability estimated by ARV is a predictor of poor short-term outcomes in a prospective cohort of minor ischemic stroke
Source: PLoS One. 2018 Aug 24;13(8):e0202317. doi: 10.1371/journal.pone.0202317 (PMC6108465; doi:10.1371/journal.pone.0202317)
Supplement: S1 File — Table A. Frequency data of 474 cases of ischemic stroke patients Table B. Data of age, NIHSS (24hours after admission) and biomarkers. Table C. Data of time from onset to admission. Table D. Data of first Blood pressure after admission. Table E. Data of Blood pressure variability (24hours after admission). Table F. Data of Blood pressure variability (D2 to D7 after admission). (DOCX) [file pone.0202317.s001.docx]

**Supplying information:**

**Table A. Frequency data of 474 cases of ischemic stroke patients**

| Characters | Frequency (n) | | Percentage (%) | Valid percentage(%) | Cumulative percentage(%) |
| --- | --- | --- | --- | --- | --- |
| Sex | male | 307 | 68.7 | 68.7 | 68.7 |
|  | female | 140 | 31.3 | 31.3 | 100.0 |
| Age | <60 | 168 | 37.6 | 37.6 | 37.6 |
|  | ≥60 | 279 | 62.4 | 62.4 | 100.0 |
| Smoke | no | 302 | 67.6 | 67.6 | 67.6 |
|  | yes | 145 | 32.4 | 32.4 | 100.0 |
| Alcohol | no | 391 | 87.5 | 87.5 | 87.5 |
|  | yes | 56 | 12.5 | 12.5 | 100.0 |
| CHD history | no | 355 | 79.4 | 79.4 | 79.4 |
|  | yes | 92 | 20.6 | 20.6 | 100.0 |
| Atrial fibrillation history | no | 413 | 92.4 | 92.4 | 92.4 |
|  | yes | 34 | 7.6 | 7.6 | 100.0 |
| Diabetes history | no | 341 | 76.3 | 76.3 | 76.3 |
|  | yes | 106 | 23.7 | 23.7 | 100.0 |
| Hypertension history^a^ | no | 167 | 37.4 | 37.4 | 37.4 |
|  | yes | 279 | 62.4 | 62.6 | 100.0 |
| TIA | no | 439 | 98.2 | 98.2 | 98.2 |
|  | yes | 8 | 1.8 | 1.8 | 100.0 |
| Antihypertensive therapy^b^ | no | 399 | 89.3 | 89.5 | 89.5 |
|  | yes | 47 | 10.5 | 10.5 | 100.0 |
| Thrombolytic therapy | no | 402 | 89.9 | 89.9 | 89.9 |
|  | yes | 45 | 10.1 | 10.1 | 100.0 |
| Atrovastatin dosage^c^ | 10mg | 82 | 18.3 | 18.8 | 18.8 |
|  | 20mg | 227 | 50.8 | 51.9 | 70.7 |
|  | 40mg | 59 | 13.2 | 13.5 | 84.2 |
|  | 80mg | 63 | 14.1 | 14.4 | 98.6 |
|  | None | 6 | 1.3 | 1.4 | 100.0 |
| Hypoglycemic therapy | no | 337 | 75.4 | 75.4 | 75.4 |
|  | yes | 110 | 24.6 | 24.6 | 100.0 |
| Type of stroke^d^ | Ischemic stroke | 447 | 99.1 | 99.1 | 99.1 |
|  | Hemorrhage stroke | 4 | 0.9 | 0.9 | 0.9 |

a, Among 474 cases of ischemic stroke patients, 1 case with unclear hypertension history

b, Among 474 cases of ischemic stroke patients 1 case was absent of antihyptensive therapy

c, Among 474 cases of ischemic stroke patients 10 cases with unknown status of atrovastatin therapy,

d, 4 hemorrhage stroke cases were found in 451cases of stroke patients.

**Table B. Data of age, NIHSS (24hours after admission) and biomarkers**

|  | Valid(n) | Absence(n) | Average value | Mid-value | SD |
| --- | --- | --- | --- | --- | --- |
| Age | 447 | 0 | 63.8 | 64.0 | 12.9 |
| NIHSS(within 24h after admission | 447 | 0 | 6.0 | 4.0 | 6.9 |
| CHO | 441 | 6 | 5.0 | 4.9 | 1.8 |
| TG | 442 | 5 | 1.7 | 1.4 | 1.2 |
| LDL | 442 | 5 | 3.0 | 3.0 | 1.4 |
| HDL | 442 | 5 | 1.2 | 1.1 | 0.7 |
| CRP | 398 | 49 | 14.6 | 3.8 | 36.1 |
| FBS | 437 | 10 | 6.7 | 5.6 | 3.1 |
| HbA1C | 411 | 36 | 6.5 | 5.8 | 1.8 |
| D-DIMER | 398 | 49 | 1408.2 | 648.0 | 2342.5 |

NIHSS: national institute of health stroke scale; CHO, cholesterol; TG, triglyceride; LDL, low density lipoprotein cholesterol; HDL, high density lipoprotein cholesterol; CRP, c reaction protein; FBS, fast blood sugar; HbA1C, hemoglobin A1C;

The unit of CHO, TG, LDL, FBS in this table is mmol/l. The unit of D-DIMER in this table is ng/ml. The unit of CRP in this table is mg/l.

**Table C. Data of time from onset to admission**

| Time to onset | Frequency (n) | Percentage (%) |
| --- | --- | --- |
| Within 12 hours | 216 | 48.3 |
| 12~23 hours | 61 | 13.6 |
| 24~35 hours | 29 | 6.5 |
| 36~47 hours | 25 | 5.6 |
| 48~71 hours | 32 | 7.2 |
| 72~168 hours | 83 | 18.6 |
| total^a^ | 446 | 99.8 |

a, 1 case was absence of detailed onset time line.

**Section 2: Blood pressure data**

**Table D. Data of first Blood pressure after admission**

| Blood pressure | Valid(n) | Absence(n) | Mean value (mmHg) | SD |
| --- | --- | --- | --- | --- |
| First SBP after admission | 447 | 0 | 155.1 | 24.9 |
| First DBP after admission | 447 | 0 | 87.1 | 16.1 |

**Table E. Data of Blood pressure variability (24hours after admission)**

| Blood pressure variability | Valid(n) | Absence(n) | Mean value (mmHg) | SD |
| --- | --- | --- | --- | --- |
| SBP SD（24h） | 447 | 0 | 13.2 | 5.3 |
| SBP CV（24h） | 447 | 0 | 0.1 | 0.4 |
| SBP ARV（24h） | 447 | 0 | 11.2 | 4.5 |
| DBP SD（24h） | 447 | 0 | 0.12 | 0.1 |
| DBP CV（24h） | 447 | 0 | 0.1 | 0.1 |
| DBP ARV（24h） | 447 | 0 | 8.4 | 3.4 |

**Table F. Data of Blood pressure variability (D2 to D7 after admission)**

| Blood pressure variability | Valid | Absence | Mean value (mmHg) | SD |
| --- | --- | --- | --- | --- |
| SBP SD（7days） | 447 | 0 | 14.3 | 4.2 |
| SBP CV（7days） | 447 | 0 | 0.1 | 0.0 |
| SBP ARV（7 days） | 446 | 0 | 13.0 | 3.8 |
| DBP SD（7 days） | 447 | 0 | 9.2 | 2.9 |
| DBP CV（7 days） | 447 | 0 | 0.1 | 0.0 |
| DBP ARV（7 days） | 446 | 1 | 8.8 | 2.5 |
